# Multi-Omic Biogeography of the Gastrointestinal Microbiota of a Pre-Weaned Lamb

**DOI:** 10.3390/proteomes5040036

**Published:** 2017-12-18

**Authors:** Antonio Palomba, Alessandro Tanca, Cristina Fraumene, Marcello Abbondio, Francesco Fancello, Alberto Stanislao Atzori, Sergio Uzzau

**Affiliations:** 1 Porto Conte Ricerche, Science and Technology Park of Sardinia, Tramariglio, 07041 Alghero, Italy; palomba@portocontericerche.it (A.P.); tanca@portocontericerche.it (A.T.); fraumene@portocontericerche.it (C.F.); 2 Department of Biomedical Sciences, University of Sassari, 07100 Sassari, Italy; mabbondio@uniss.it; 3 Department of Agricultural Science, University of Sassari, 07100 Sassari, Italy; fancello@uniss.it (F.F.); asatzori@uniss.it (A.S.A.)

**Keywords:** metaproteomics, microbial community, mucosa, ruminant, sheep

## Abstract

The digestive functions of the pre-weaned lamb gastrointestinal tracts (GITs) have been the subject of much research in recent years, but the microbial and host functions underlying these complex processes remain largely unknown. Here, we undertook a proof-of-principle metaproteogenomic investigation on luminal and mucosal samples collected from 10 GITs of a 30-day-old pre-weaned lamb. We demonstrate that the analysis of the diverse ecological niches along the GITs can reveal microbiota composition and metabolic functions, although low amounts of microbial proteins could be identified in the small intestinal and mucosal samples. Our data suggest that a 30-day lamb has already developed mature microbial functions in the forestomachs, while the effect of the milky diet appears to be more evident in the remaining GITs. We also report the distribution and the relative abundance of the host functions, active at the GIT level, with a special focus on those involved in digestive processes. In conclusion, this pilot study supports the suitability of a metaproteogenomic approach to the characterization of microbial and host functions of the lamb GITs, opening the way to further studies aimed at investigating the impact of early dietary interventions on the GIT microbiota of small ruminants.

## 1. Introduction

In mammals, the gut microbiota has been clearly demonstrated to be of paramount importance for its roles in digestive processes, nutrient absorption and metabolism, immune response, and gastrointestinal tissues development. Noteworthy, microbial communities that colonize the different gastrointestinal tracts (GIT) have to be regarded as diverse ecological systems whose taxonomies and metabolic activities strongly depend on the specific digestive tract environments, at the intimate interface with the mucosa or within the luminal milieu. Compared to monogastric animals, sheep and other ruminants possess further complexity in their GIT microbiota, given the extraordinary digestive and metabolic role of the microorganisms at the forestomach sites (rumen, reticulum and omasum). Here, cellulolytic microbes break down cellulose and fibrous compounds, which are abundant in forage-based diets, whereas amylolytic microbes ferment starch and sugars, and both produce volatile fatty acids (VFAs). VFAs (such as acetate, propionate and butyrate) are primarily produced from dietary carbohydrates through a large variety of metabolic pathways and provide about 70% of the ruminant’s energy supply, being directly oxidized or acting as key precursors to liver gluconeogenesis (propionate) or lipid biosynthesis (acetate). In addition, the feed-fermenting microorganisms (Bacteria, Archaea, Fungi and Protozoa) growing in the sheep rumen are the major source of proteins, partly hydrolyzed in the gastric stomach, and of amino acids that flow to the small intestine.

In the postnatal life, from birth to weaning, the lamb is defined as a functional monogastric, since the rumen is not developed and most of the nutrients are derived from enzymatic processes occurring into the gastric stomach and intestine [1,2]. A broad number of studies have been conducted in newborn and pre-weaned lambs, but few of them were recently focused on rumen microbiota taxonomy [3,4,5]. Noteworthy, dietary interventions in pre-weaned lambs were reported to affect significantly the quality of microbial colonization and functions involved in further rumen development. Specifically, addition of linseed oil to the lamb meal modified the long-term structures of microbial communities (especially Bacteria and Archaea), even without feed intake or weight gain variations [5]. These studies highlight the importance of applying short-term dietary changes (early in life) to manipulate the rumen microbiota and to obtain (later in adult life) an improved response to a defined diet, ameliorating feed efficiency and/or other productive performances. In this sense, it is fundamental to know the most suitable postnatal windows to implement effective dietary interventions enabling metabolic imprinting and programming [6,7]. Early solid feed intake seems to favor microbial colonization and early functional establishment of the different GITs [8,9,10]. In addition, glucose and lipid metabolism (especially insulin secretion, adipose tissue sensitivity to insulin and uptake of glucose) in mature sheep are highly affected by diet and nutritional status experienced in prenatal and postnatal phases, as reviewed by Khanal et al. [11], suggesting that functional changes in these phases are fundamental to permanent metabolic programming and nutrient partitioning. 

In addition to microbial communities organization (taxonomy), new studies are required to extend knowledge of the microbial functions during rumen early development. Recently, the microbial molecular mechanisms of rumen development have been analyzed in 6-week-old lambs, by means of transcriptomics, revealing that the rumen microbiota was responsive to early administration of starter feed, and showing significant earlier development of rumen metabolism compared to the milk-fed animals [12].

As GIT microbiota fermentation capability appears to be of vital importance to the animal nutrition since early life, the knowledge of the combined effect of dietary pattern and microbiota in pre-weaned animals are certainly key to the ensuing sheep production performances. Thus, in addition to the rumen development, a more integrate and holistic perspective should be adopted to investigate the development of microbial communities taxonomy and metabolism along other intestinal tracts, given the meaningful relevance of the whole GIT on digestion and metabolism [13,14]. Concerning the GIT distal to the forestomach, from the abomasum to the rectal ampulla, however, no studies have been yet undertaken to measure the microbial functions actively expressed by the microbiota in the pre-weaned lamb. At this age, the GIT microbiota is expected to be shaped by the dam’s teat and the environmental microbiota, milk diet, starters and initial grazing, together with the co-development of the lamb digestive functions. To this end, proteomics might enable the simultaneous investigation of the microbial and host digestive functions throughout the lamb GIT. Noteworthy, the tracts of the abomasum and the small intestine represent harsh environments for microbial survival and replication, due to physical and chemical conditions (lowered pH, bile salts and pancreatic secretions). Therefore, the lower bacterial load poses technical limitation to investigate the microbial metabolic functions at the abomasum, duodenum, jejunum, and the proximal ileum level.

Hence, in this study, we aimed to investigate the composition and the functions actively expressed by the microbiota associated to the GIT of a pre-weaned 30 days lamb. Through a metaproteogenomic approach, we provide here an extensive description of the different microbial communities colonizing 10 tracts of a pre-weaned lamb GIT, from the rumen to the rectum, and of the host digestive functions in the different GIT biotopes.

## 2. Materials and Methods

### 2.1. Animal Description and Sample Collection

A lamb of Sarda dairy sheep was slaughtered at 30 days after birth. The lamb was fed with mother’s milk, although traces of corn grain, beet pulp and ryegrass hay may have been ingested, as it was kept in a confined overgrazed grass pasture with free access to the mother’s diet. Immediately after slaughter ten different GITs (rumen, reticulum, omasum, abomasum, duodenum, jejunum, ileum, cecum, colon, and rectum) were collected. At necropsy, each tract was isolated with stitches to avoid the loss of the luminal content, cut, gently washed externally with saline solution, then immediately frozen and stored at −80 °C until use. Concerning the longest intestinal tracts, such as duodenum and jejunum, only the central section (measuring approximately 10–15 cm) was kept. 

Luminal and mucosal contents were collected from GITs as follows: each tract was thawed at 4 °C, washed externally with saline solution, unrolled and opened by cutting an extremity on a clean petri dish to collect the possibly leaking luminal fluid. When contents were liquid, the external wall was “squeezed” by flattening with a glass slide. In case of too little amount of luminal material for the subsequent analyses (omasum, ileum and cecum), the inner part was delicately washed with saline and the obtained luminal rinse was collected. Moreover, as abomasum, colon and rectum presented a more compact content, a central portion of the solid matter was collected. Then, residual luminal material was carefully washed out with saline and tracts were opened longitudinally and stretched on a sterile petri dish to collect mucosal content by scraping with a glass slide. All luminal (n = 10) and mucosal (n = 10) samples were divided into two portions, for DNA and protein extraction, respectively.

All the experimental procedures carried out on the animal were in accordance with the EC Council Directive guidelines on the protection of animals used for scientific purposes (Directive 2010/63/EU of the European Parliament and of the Council of 22 September 2010) and were approved by the Ethical Committee for animal welfare (OPBSA) of the University of Sassari.

### 2.2. DNA Sample Preparation and Sequencing

Luminal and mucosal samples were subjected to DNA extraction using PowerSoil^®^ DNA Isolation Kit (Mo Bio Laboratory Inc., now part of Qiagen, Carlsbad, CA, USA), following the manufacturer’s instruction. We performed a full-length 16S rRNA gene amplification using the universal primers 27F and 1492R (AGAGTTTGATCMTGGCTCAG and TACGGYTACCTTGTTACGACTT, respectively) and cleaned up using AMPure XP (Beckman Coulter, Brea, CA, USA) magnetic beads. Then, a nested amplification of the V4 region of 16S rDNA was performed using purified16S rRNA gene amplification. To amplify V4 region, we used primers 515F and 806R (GTGCCAGCMGCCGCGGTAA and GGACTACHVGGGTWTCTAAT, respectively) modified to contain adaptors for MiSeq sequencing. V4 libraries were constructed using Illumina’s recommendations, as implemented in 16S Metagenomic Sequencing Library Preparation guide, and DNA sequencing was performed in duplicate with the MiSeq sequencer (Illumina, San Diego, CA, USA), using the MiSeq Reagent Kit v3 according to the manufacturer’s specifications to generate paired-end reads of 201 bases in length in each direction. Raw read sequences were deposited in the European Nucleotide Archive under the Project Accession Number PRJEB23436. The paired-end reads, with a minimum overlap of eight bases, were merged using the script join_paired_ends.py included in the QIIME package [15], retaining only reads with a length >200 bps for further analysis. Generation of operational taxonomic units (OTUs) and their taxonomy assignment were done on all samples according to a previously described pipeline [16]. The relative proportion of read counts was used as a quantitative estimation of the abundance of each taxon, after aggregation of count data at phylum and family taxonomic levels.

Additionally, 15 metagenomes selected from luminal and mucosal samples were sequenced, in order to create a custom sequence database for metaproteomic analyses. DNA libraries were constructed with the Nextera XT kit (Illumina), and sequenced with the MiSeq sequencer, using the MiSeq Reagent Kit v3, with the paired-end method and 201 cycles of sequencing.

### 2.3. Protein Sample Preparation and Metaproteome Analysis

Luminal and mucosal samples were subjected to a previously reported protein extraction procedure [17]. Briefly, samples were resuspended in an SDS-based, reducing extraction buffer and subjected to bead beating combined with freeze-heating. Extracted proteins were cleaned up, alkylated and digested with trypsin according to the filter-aided sample preparation (FASP) procedure [18], with minor modifications illustrated elsewhere [19,20].

LC-MS/MS analysis was carried out using an LTQ-Orbitrap Velos mass spectrometer (Thermo Fisher Scientific, Waltham, MA, USA) interfaced with an UltiMate 3000 RSLCnano LC system (Thermo Fisher Scientific). The single-run 1D LC peptide separation was performed as illustrated earlier [17], loading 4 μg of the peptide mixture obtained per each sample and applying a 247 min separation gradient. The mass spectrometer was set up in a data-dependent MS/MS mode, with Higher Energy Collision Dissociation as the fragmentation method, as described previously [19]. Peptide identification was performed using Proteome Discoverer (Thermo Fisher Scientific, version 1.4), with Sequest-HT as search engine and Percolator for peptide validation (FDR < 1%). Search parameters were set as described previously [20].

For each sample, parallel searches were performed using three different sequence databases. The first database was composed by the metagenomic sequences obtained in this study (3,474,764 sequences). Paired reads were merged, filtered and clustered using USEARCH (version 8.0.1623) [21], with previously illustrated parameters [16]. In parallel, raw reads were also assembled into contigs using MetaVelvet (version 1.2.01) [22], with 61 as k-mer length, 200 as insert length and 300 as minimum contig length. Finally, FragGeneScan (version 1.30) [23] was used to find open reading frames, with the training for Illumina sequencing reads with about 0.5% error rate.

The second database was a selection of public sequences based on 16S metagenomics analysis outputs (24,350,176 sequences). In detail, all sequences belonging to a microbial genus whose relative abundance was >0.1% in at least one of the samples were selected and downloaded from UniProtKB (2017_07 update). Metaproteomic data were obtained by merging results of searches against the two above mentioned databases. The FASTA file containing all database sequences identified by metaproteomics was subjected to taxonomic and functional annotation using MEGAN (Community Edition, version 6.9) [24], after DIAMOND (version 0.8.22) [25] search against the NCBI-nr DB (2016/09 update) using the blastp command with default parameters. InterPro module embedded in MEGAN was selected as source for functional annotation. An additional annotation concerning metabolic pathways was accomplished by aligning the identified protein sequences against a database containing all bacterial sequences from UniProtKB/Swiss-Prot (release 2017_09) using DIAMOND (blastp module, e-value threshold 10^−5^) and retrieving pathway information via the ‘retrieve’ module of the UniProtKB website by providing the UniProtKB/Swiss-Prot accession numbers [26].

Finally, a third database (UniProtKB sequences belonging to the suborder Ruminantia, 2017_10 update, 115,553 sequences) was employed to achieve information concerning the host.

The mass spectrometry proteomics data have been deposited to the ProteomeXchange Consortium via the PRIDE [27] partner repository with the dataset identifier PXD008192.

### 2.4. Data Analysis and Graph Generation

Richness and alpha diversity (Shannon index) were calculated for 16S data (taxonomic family level), metaproteomic data (functions) and host proteome data. Line and bar graphs were created using GraphPad Prism (version 5.03, GraphPad Software Inc., La Jolla, CA, USA) starting from relative abundance data. Principal component analysis (PCA) plots were generated using Perseus (version 1.5.1.6) [28] starting from relative abundance data. The heatmap was generated using the web application Morpheus (https://software.broadinstitute.org/morpheus/), starting from relative abundance data. Differential analysis was performed using an established test for count data, based on a beta binomial model and an inverted beta binomial model for unpaired and paired data, respectively [29,30]. Adjustment for multiple testing was carried out according to Benjamini and Hochberg [31].

## 3. Results and Discussion

### 3.1. Experimental Design and General Metrics of Omic Data

Noteworthy, along each pre-weaned lamb GIT, microbial communities are expected to vary according to specific and dynamic combinations of dietary patterns, host enzymes, metabolites, and immunological effectors. In turn, the microbial antigenic repertoire and their functional arsenal are expected to maintain the intestinal homeostasis in each GIT. With the aim of achieving knowledge of both taxonomy and functions of the different GIT microbiotas in the pre-weaned lamb and, to this end, to investigate the feasibility of a metaproteogenomic approach, 10 GITs (rumen, reticulum, omasum, abomasum, duodenum, jejunum, ileum, cecum, colon, and rectum) were collected from a 30-day-old Sarda lamb fed mother’s milk and forage. In view of the large number of tracts to be analyzed and of the multi-omic approach selected, we chose to perform this proof-of-principle study on a single animal. All luminal (n = 10) and mucosal (n = 10) samples were divided into two portions, for DNA and protein extraction, respectively.

DNA extracted from luminal and mucosal contents was first directly subjected to amplification of the V4 hypervariable region of the 16S rRNA gene. However, the majority of the DNA samples did not provide evidence of bacterial PCR products when checked on 2% agarose gel (data not shown), possibly due to the lowest microbial genomic contents. In view of this, we decided to perform a full-length 16S rRNA gene amplification, which was able to reach a much higher amplification yield. Then, a nested amplification was devised, comprising V4 amplification on full-length 16S amplicons, which led to obtain a satisfactory amount of PCR products for all samples. Therefore, we decided to employ the nested amplification approach, although being aware of the possible biases that this method can generate with respect to quantitative ratios among microbial species. Amplicons were subsequently subjected to deep sequencing with a MiSeq Illumina sequencer (1,147,915 reads in total; the number of reads obtained per each sample are listed in Table S1). For the sake of brevity, data concerning the sequencing of nested rRNA gene amplicons (V4 region on full-length 16S) will be hereafter referred to as “16S”. Additionally, a selection of 15 whole metagenomes were sequenced, with the purpose of creating a custom sequence database for metaproteomic analyses.

In parallel, proteins were extracted from luminal and mucosal samples, processed according to the FASP procedure, and finally analyzed by high-resolution LC-MS/MS. Multiple database search strategies were employed to maximize microbial and host protein identification, according to previous reports from ours and other groups [32,33,34,35]. Specifically concerning the microbial identifications, the first database was composed by the metagenomic sequences (raw and assembled) obtained in this study, while the second was a selection of UniProtKB sequences belonging to the microbial genera identified through 16S metagenomics. A total of 528,572 peptide-spectrum matches (PSMs), of which 412,940 related to the host and 115,632 classified as microbial, were identified. As reported in Table S2, a low depth of microbial-related information was reached for some GITs, especially those belonging to the small intestine and mucosal samples in general.

**Figure 1:**
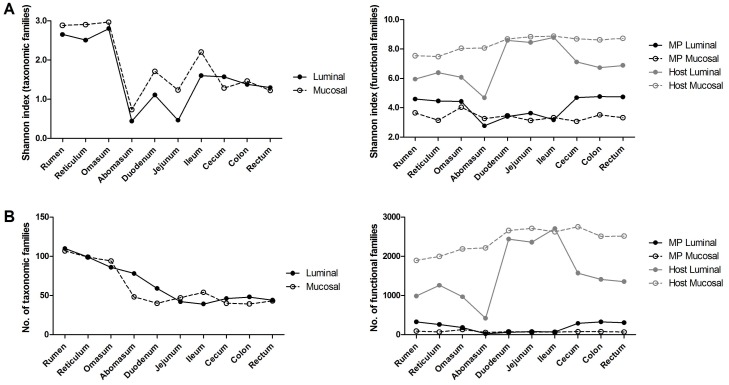
Alpha-diversity and richness distribution along the gastrointestinal tracts of a pre-weaned lamb. (**A**) Line graphs illustrating Shannon index values (alpha-diversity) computed based on taxonomic families (left), according to 16S data, and both host and microbial functional families (right), according to metaproteomics data, for each luminal and mucosal gastrointestinal sample. (**B**) Same as (**A**), but concerning richness (number of observed taxonomic families, left, and both host and microbial functional families, right).

Richness and alpha-diversity of taxonomic and functional data concerning the lamb GITs are showed in Figure 1. As illustrated by the line graphs, the highest values of taxonomic alpha-diversity and richness were observed in the forestomachs, while a remarkable drop was observed in the abomasum and in all the intestinal tracts, with a slight increase of the richness in the large intestine; luminal and mucosal samples showed comparable trends. Concerning the number and complexity of microbial functions (functional families), those detected at the mucosa interface appeared consistent throughout the GITs, while those associated to the digesta were higher in the tracts with higher fermentative activity (i.e., forestomachs and large intestine). The complexity of the microbiota and its functions, therefore, is in keeping with the massive fermentation capability to be reached in the rumen, reticulum and omasum, and with the significant contribution to feed fermentation provided also by microorganisms that colonize the colon, as recently reported [4,16]. The harsher conditions of the abomasum and the duodenum, due to the gastric secretion in the former and the bile and pancreatic secretion in the latter, might explain the drop of microbiota richness and alpha diversity in the abomasum and the small intestine. Furthermore, when considering functional data, richness and diversity of host protein functions were higher than those of microbial functions along all GITs, with a remarkable increase in the small intestine where the microbial function exhibited the lowest values.

**Figure 2:**
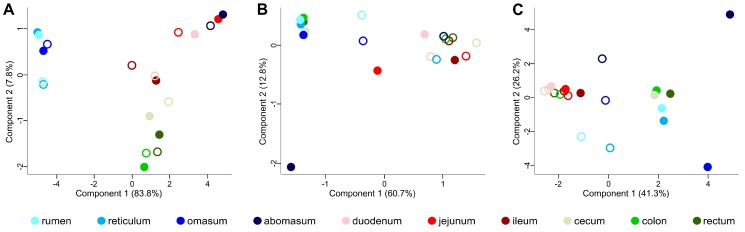
Beta-diversity at taxonomic and functional level within the gastrointestinal tracts of a pre-weaned lamb. PCA plots were generated starting from the relative abundance of taxonomic families according to 16S data (**A**), microbial functional families (**B**), and host functional families (**C**), according to metaproteomic data. Each dot indicates a sample, while each color corresponds to a different gastrointestinal tract, with color-filled dots corresponding to luminal and empty dots to mucosal samples.

Beta-diversity was also evaluated through PCA plots (Figure 2). The plot based on 16S taxonomic data showed a clear separation between forestomachs and the other GITs according to the first component; in addition, small and large intestine clustered separately according to the second component. No clear separation among luminal and mucosal samples could be observed (Figure 2A). According to metaproteomic data, a global separation was seen between luminal contents of forestomachs and large intestine on one hand, and luminal contents of small intestine and all mucosal contents on the other hand (Figure 2B). According to these data, similar fibrolytic taxa are expected to be active in the lumen of the forestomachs and the larger intestine. Furthermore, abomasum lumen was clearly different from the other samples based on the second component. Noteworthy, abomasum, duodenum and jejunum showed low diversity at taxonomic level (Figure 2A). On the other hand, at functional level, abomasum showed high diversity compared to duodenum and jejunum (Figure 2B), suggesting that the same assortment of microorganism is functionally divergent, possibly as a consequence of the stressful exposure to lowest pH in the former tract. Similar results were found when considering host proteins (Figure 2C).

### 3.2. Taxonomic Distribution of the Microbiota along the Gastrointestinal Tract of a Pre-Weaned Lamb

**Figure 3:**
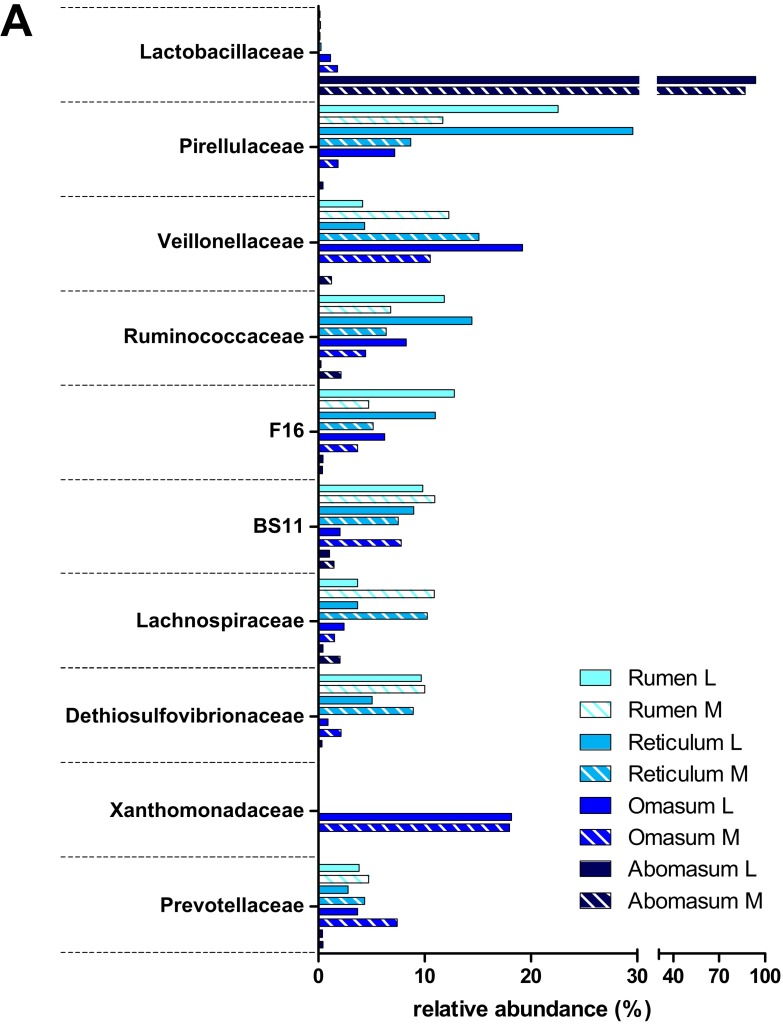

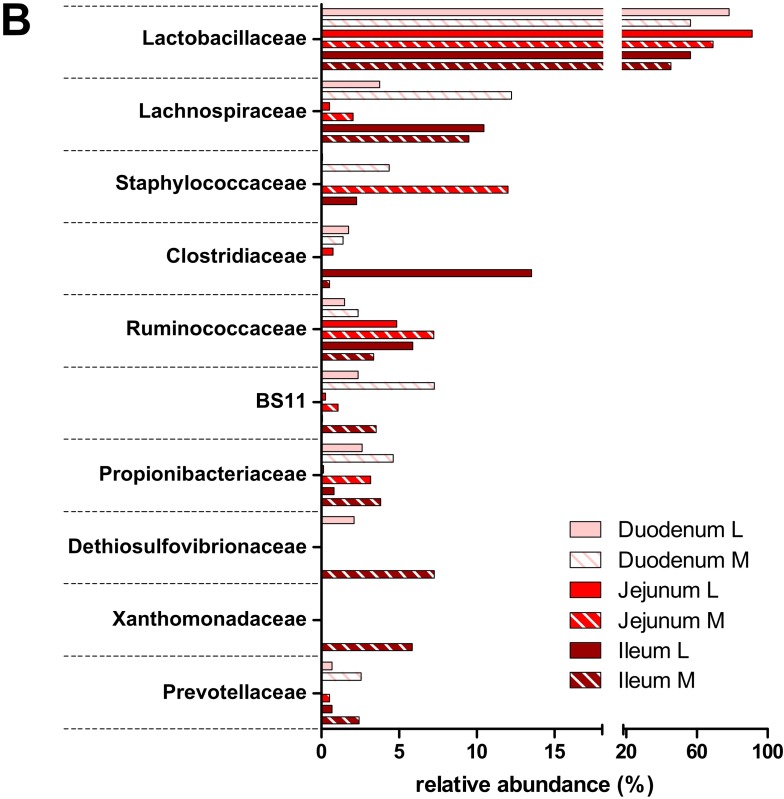

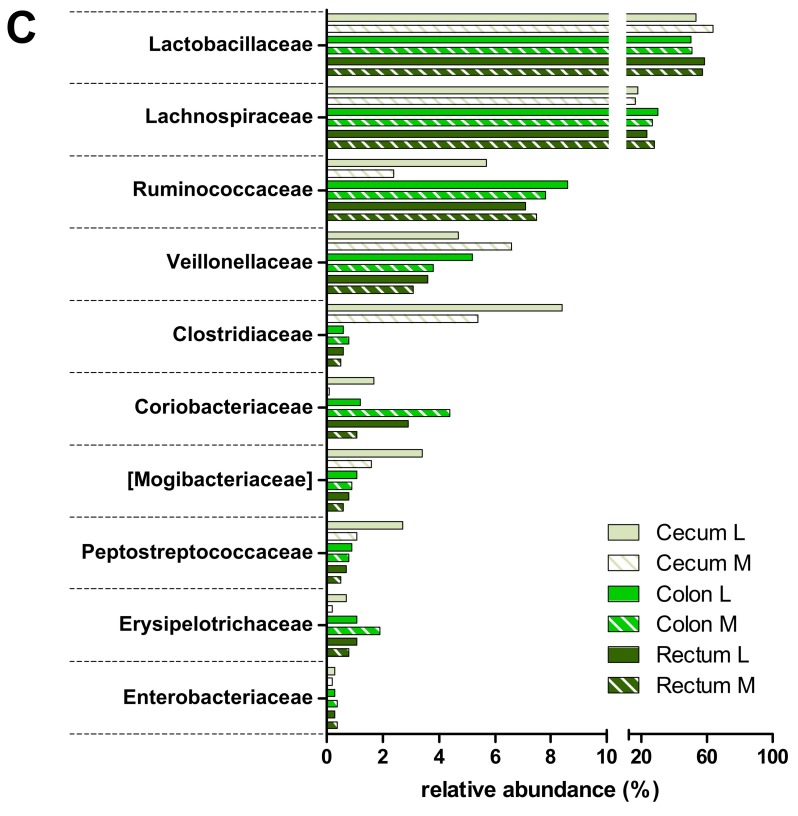
Top 10 most abundant taxonomic families along the gastrointestinal tracts of a pre-weaned lamb. The relative abundance of microbial families identified in the forestomach (**A**), small intestinal (**B**), and large intestinal (**C**) tracts according to 16S data is reported. L, luminal; M, mucosal.

Focusing on taxonomy, important differences were detected along the GITs of the pre-weaned lamb under study. At total of 37 bacterial and archaeal phyla (Dataset S1) were detected. Firmicutes and Bacteroidetes were the most abundant phyla in all GITs consistently with previous reports from other authors on the adult sheep [12,16,36]. Figure 3 reports the top 10 microbial families and their relative abundances, computed by aggregating OTUs at family level.

At first glance, a strong divergence was observed between the forestomachs and all the other tracts, in accordance with alpha- and beta-diversity. As depicted in Figure 3A, rumen and reticulum showed high levels of Pirellulaceae (belonging to the phylum Planctomycetes; 22% and 30%, respectively), Ruminococcaceae (phylum Firmicutes; 12% and 14%), F16 (phylum TM7; 13% and 11%), BS11 (phylum Bacteroidetes; 10% and 9%) and Dethiosulfovibrionaceae (phylum Synergistetes; 10% and 5%) in the lumen, plus several other families with a moderate relative abundance (>3%). Planctomycetes, forming the PVC superphylum together with Verrucomicrobia and Chlamydiae [37], belong to the usual components of the ruminal microbiota of sheep [38], cattle [39] and dairy cows [40]. Specifically, the family Pirellulaceae was found in a greater extent in this work than in the rumen microbiota of mature sheep, cattle and deer [41]. The role of Planctomycetes in the GIT is still unclear, although anaerobic sulfur reduction and sugar fermentation have been suggested as two possible metabolic processes enabling Planctomycetes to grow in anaerobic environments [42]. The Bacteroidetes family BS11 is able to ferment hemicellulosic sugar to VFAs, which are considered essential to ruminant energy supply [43]. The composition of the mucosal microbiota inhabiting the pre-weaned lamb forestomachs appeared to be globally comparable to that found in the lumen, in spite of a drop of Pirellulaceae counterbalanced by higher levels of Veillonellaceae (phylum Firmicutes; 12% in rumen and 15% in reticulum) and Lachnospiraceae (phylum Firmicutes; both 11%). Furthermore, Veillonellaceae (19% in the luminal and 11% in the mucosal community) and Xanthomonadaceae (phylum Proteobacteria; both 18%) were the most abundant families observed in the omasum microbiota, where some families observed in the first two tracts were also detected (such as Ruminococcaceae, Pirellulaceae and F16). Veillonellaceae and Lachnospiraceae have been recently identified as part of the core rumen microbiome in cattle [44]. The Veillonellaceae family produces propionate as a major fermentation product, while Lachnospiraceae synthesize butyrate that plays an important role in rumen development [8]. Several members of the Xanthomonadaceae are able to produce xylanases, and therefore to ferment xylan, a major structural component of plant cell wall. This may make them capable to survive in anaerobic environments, as the omasum; furthermore, members of Proteobacteria might play a role in scavenging oxygen diffusing from the capillary system, creating the condition for the establishment of anaerobic communities [6,45,46]. Considering both luminal and mucosal microbial communities, rumen, reticulum and omasum presented a well-assorted microbiota, with several families overtaking the 3% of their relatives abundance. In addition, similar values of Firmicutes/Bacteroidetes (F/B) ratio were observed in the luminal and mucosal content of rumen and reticulum (0.77 vs. 0.95, and 0.94 vs. 1.13), while a higher F/B ratio was detected in the luminal content of omasum compared to the mucosal microbiota (2.46 vs. 0.82) (Dataset S1). Archaea families were also well represented (around 1% and up to 3% in the omasum) (Dataset S2). 

The remaining seven GITs showed a completely different structure (Figure 3B,C), with Lactobacillaceae (phylum Firmicutes) being the most predominant (and almost exclusive) family in the abomasum (94% lumen and 87% mucosa) and gradually decreasing along the intestine, reaching a minimum of 46% in the ileal mucosal microbiota, and remaining around 50% in the large intestine tracts, in line with the diet still mainly based on milk. This also affected the F/B ratio, clearly unbalanced towards Firmicutes. Other Firmicutes families exhibiting a remarkable abundance in the gut were Lachnospiraceae (duodenal mucosa 12%, ileum, 10%, and up to 29% in colonic lumen), Clostridiaceae (13% and 8% in the lumen of ileum and cecum, respectively) and Ruminococcaceae (around 8% in colon and rectum). With the exception of Lactobacillaceae, these families were predominant also in the adult sheep, followed by Veillonellaceae and Mogibacteriaceae; compared to the adult animal, the most striking difference was the very much lower abundance of families belonging to Bacteroidetes and Verrucomicrobia [16]. Such differences might be explained with a lower complexity of dietary glycans escaping the rumen in the pre-weaning, since this heterogeneous group of molecules is known to shape the composition of the gut microbiota [47]. Finally, very few Archaea were detected, consistently with a lower methanogenic activity compared to lamb forestomachs and to large intestine in the adult sheep (Datasets S1 and S2). Indeed, the syntrophic relationships between cellulolytic, H_2_-producing bacteria (Ruminococcaceae, Lachnospiraceae, etc.) and H_2_-consuming methanogenic archaea, commonly observed in the rumen, might be displaced here by the capability of Lactobacillaceae to inhibit hydrogen fermentation by substrate competition (lactic acid fermentation).

### 3.3. Functions and Metabolic Pathways of the Pre-Weaned Lamb Gastrointestinal Microbiota

**Figure 4:**
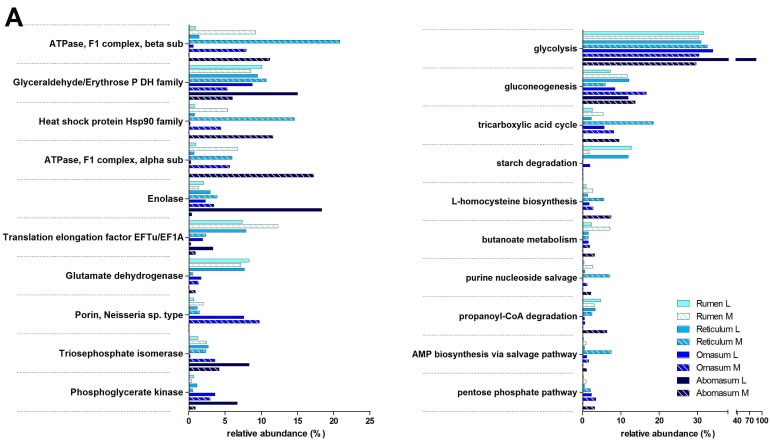

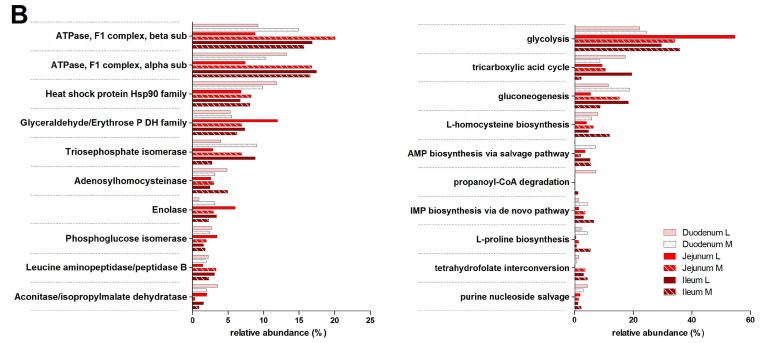

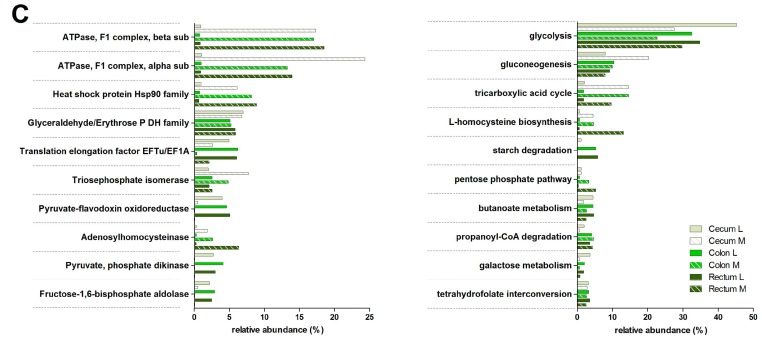
Top 10 most abundant microbial functions and metabolic pathways along the gastrointestinal tracts of a pre-weaned lamb. The relative abundance of microbial functions (left) and metabolic pathways (right) identified in the forestomach (**A**), small intestinal (**B**), and large intestinal (**C**) tracts according to metaproteomic data is reported. L, luminal; M, mucosal; P, phosphate; DH, dehydrogenase; sub, subunit.

As illustrated in Figure 4, several enzymes involved in glycolysis and pyruvate metabolism, as well as ribosomal proteins, were detected in the top 10 microbial functions upon metaproteomic analysis (the InterPro database was selected for functional annotation, while assignment to metabolic pathways was based on UniProtKB annotation). Glutamate dehydrogenase overtook the 8% of relative abundance in rumen and reticulum, dropping to much lower percentages in the small (<1%) and large intestine (<2%). Phosphoserine aminotransferase exhibited a similar trend. These findings are in line with the known role of these enzymes in several catabolic and anabolic pathways of amino acid metabolism, consistently with the intense microbial proliferation in these sites.

According to pathway classification (Figure 4 right), proteins involved in starch degradation were almost exclusively detected in the luminal samples from rumen, reticulum, colon and rectum, consistently with the known biogeography of fiber fermentation. The presence of enzymes devoted to starch degradation in the lower GITs is in agreement with the mentioned ingestion of small pieces of corn grain from the mother’s diet in early postnatal phases, even before the first stages of rumen development. Nonetheless, up to 50% of the starch may escape to the small intestine also in mature ruminants [48], where the hindgut may thus avoid the inefficiencies of rumen fermentation [49]. This phenomenon has been demonstrated to be highly dependent on the diet and in particular on the presence of corn, sorghum, and legumes [50,51].

We then focused our attention on glycoside hydrolases (GHs), in view of their key role in plant biomass degradation. Peptides belonging to ten different GH families were detected. GH 28, comprising mainly polygalacturonases digesting pectins, representing the high digestible fiber sources for ruminants [52], was present almost in all GITs, in agreement with the fact that fibrous particles ingested could bypass the forestomach, not only before the rumen development [38]. Other families were more typical of specific tracts. These include GH 9 (formerly known as cellulose E) that was only found in the ruminal lumen, GH 36 (alpha-galactosidase) higher in the omasum, and GH 2 (beta-galactosidase) present mainly in the lumen of the large intestine (Dataset S3). 

Next, given the relevant fermentative activity exerted by both rumen and colon, we sought to compare the functional assortment of the microbiota inhabiting the lumen of these two GITs, and to determine if similar/different enzymatic activities are performed by similar/different members of the microbiota in a pre-weaned lamb. As a result, methanogenic enzymes, expressed by Euryarchaeota (specifically Methanobacteriaceae), were only found in the rumen, while several proteins responsible for acetate biosynthesis (including carbon monoxide dehydrogenase and enzymes of the Wood-Ljungdahl pathway, mainly produced by Firmicutes) were exclusively detected in the colon (Datasets S3–5). Colonization of the rumen by methanogens is more competitive than that of reductive acetogens and starts in the first days of life to sustain a functional rumen by reducing hydrogen concentration [53].

Moreover, when considering proteins assigned to Ruminococcaceae, some functions, including pilus retraction protein PilT, aconitase/isopropylmalate dehydratase, enolase and flagellin, were found to be rumen-specific, while others, comprising many ribosomal proteins and enzymes involved in pyruvate and butyrate metabolism, appeared to be colon-specific.

As illustrated previously (Figure 1), there were considerable differences in the amount of microbial peptides detected based on the specific sample taken into consideration (GIT and/or lumen vs mucosa). This may have led to some biases, especially when comparing relative abundances between samples with much different identification depth. However, glyceraldehyde-3-phosphate dehydrogenase, known to be a housekeeping protein with quite constant abundance in different organisms/tissues, was consistently detected at comparable levels in samples with different in identification depth (e.g., lumen and mucosa of large intestinal tracts).

### 3.4. Host Protein Functions along the Pre-Weaned Lamb Gastrointestinal System: Focus on Digestion

**Figure 5:**
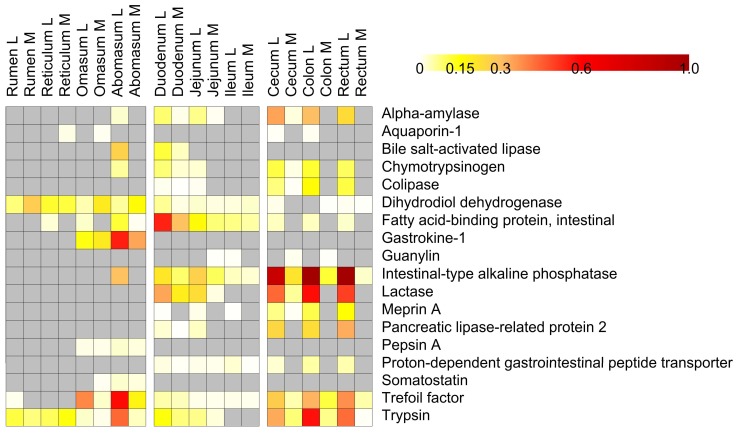
Heatmap illustrating host digestive functions distribution along the gastrointestinal tracts of a pre-weaned lamb. Columns represent samples, while rows represent functions. Grey squares correspond to no-detected features. Only functions with mean relative abundance >0.001% are shown.

Metaproteomics allows the identification of both microbial and host proteins in the same sample. Therefore, we exploited this omic approach to elucidate the distribution of lamb proteins along the different GITs. Many structural proteins were identified, especially in the mucosal samples. However, we decided to focus on host digestive enzymes, in view of their importance for animal production and management. As shown in Figure 5 (and detailed in Dataset S6), intestinal-type alkaline phosphatase, lactase, trypsin, trefoil factor, alpha-amylase, colipase and other enzymes were detected at very high levels in lumen of the various tracts of the large intestine. Previous studies confirm the high activity of these enzymes in duodenum and colon, as well as the association of this catalytic activity to GIT development stages in small ruminants [54]. It is generally assumed that lactase activity decreases with age whereas all the other hydrolytic enzymes increase reaching stable ranges at ruminal level, 30 days after birth, in dairy calves [55]. In pre-weaned lambs, fed only milk, a quite stable enzymatic hydrolytic activity in different traits of the small intestine was also observed from birth to 56 days [56].

On the other hand, in this study, gastrokine, pepsin and somatostatin were detected in the omasum and, at even higher amounts, in the abomasum. Fatty acid-binding protein was instead typical of duodenum, whereas bile salt-activated lipase was found mostly in abomasum and duodenum. 

## 4. Conclusions

The multi-omic approach proposed in this work was found to enable deep investigation of the lamb GIT microbiota and proteome. In view of the results described in this pilot study, such an approach holds promise to be applied in future studies for the investigation of microbial structure, functional establishment of digestion and metabolic processes induced by dietary regimens from birth to weaning, targeting key aspects of the metabolic programming in ruminants.

Furthermore, the data presented here may lay the methodological groundwork for research aimed at understanding the mechanisms behind early-life functional changes occurring in ruminants in response to dietary interventions, in particular elucidating the impact of microbiota-host interactions on animal production and health.

## Supplementary Materials

The following are available online at www.mdpi.com/2227-7382/5/4/36/s1, Table S1: 16S data statistics. Table S2: Metaproteomic data statistics. Dataset S1: Tables reporting identified and differential phyla from 16S data. Dataset S2: Tables reporting identified and differential taxonomic families from 16S data. Dataset S3: Tables reporting identified and differential microbial functions from metaproteomics data. Dataset S4: Tables reporting identified and differential microbial function/phylum combinations from metaproteomics data. Dataset S5: Tables reporting identified and differential microbial function/family combinations from metaproteomics data. Dataset S6: Tables reporting identified and differential host functions from metaproteomics data.

## Abbreviations

FASP	filter-aided sample preparation
F/B	Firmicutes/Bacteroidetes
FDR	false-discovery rate
GH	glycoside hydrolase
GIT	gastrointestinal tract
OTU	operational taxonomic unit
PCA	principal component analysis
PSM	peptide-spectrum matches
VFA	volatile fatty acid
